# Electromagnetic Piezoelectric Acoustic Sensor Detection of Extracellular Vesicles through Interaction with Detached Vesicle Proteins

**DOI:** 10.3390/bios10110173

**Published:** 2020-11-11

**Authors:** Loránd Románszki, Zoltán Varga, Judith Mihály, Zsófia Keresztes, Michael Thompson

**Affiliations:** 1 Institute of Materials and Environmental Chemistry, Research Centre for Natural Sciences, H-1117 Budapest, Hungary; romanszki.lorand@ttk.hu (L.R.); varga.zoltan@ttk.hu (Z.V.); mihaly.judith@ttk.hu (J.M.); 2 Department of Chemistry, University of Toronto, Toronto, ON M5S 3H6, Canada; m.thompson@utoronto.ca

**Keywords:** electromagnetic piezoelectric acoustic sensor, quartz, adsorption, diagnostics, extracellular vesicle

## Abstract

An electromagnetic piezoelectric acoustic sensor (EMPAS) was used to study the non-specific adsorption of human red blood cell-derived extracellular vesicle preparations. Vesicle storage history (temperature and duration) highly affected the obtained results: The signal change, namely the frequency decrease of the crystal measured at 20 °C, was negligibly small (<1 s^−2^) when the vesicle solutions had previously been stored at 4 °C, and was in the order of 10 s^−2^ when the vesicle solutions had been stored at −30 °C. Moreover, the rate of frequency decrease increased exponentially with the storage time at −30 °C. Upon a 4 °C storage period following the −30 °C storage period of the same sample, the measured frequency decrease dropped, suggesting a partial relaxation of the system. The results are explained by the disintegration of the vesicles triggered by the freeze–thaw cycle, likely due to the detachment of proteins from the vesicle surface as was proved by size-exclusion chromatography. Surface modification of the sensor crystal provided the possibility of signal enhancement, as the maximum rate of the frequency change for the same vesicle concentrations was higher on hydrophobic, octadecyl trichlorosilane–modified quartz than on hydrophilic, bare quartz. The EMPAS signal has been associated with the amount of detached proteins, which in turn is proportional to the originating vesicle concentration.

## 1. Introduction

Extracellular vesicles (EVs) are cell-released lipid particles containing a large variety of proteins, nucleic acids and metabolites. They offer vast biological information as a cargo in intercellular communication, and are also a potential target in diagnostic techniques [1,2]. In order to use EVs in diagnosis, well characterized sampling and separation requirements must be fulfilled. 

Guideline literature MISEV2018 [3] stresses that purity requirements must be met in order for EV isolates to provide reliable information on associated functional activity. Isolation of EVs from non-EV material and soluble non–EV-associated proteins is of high importance as the impurities may interfere with particle number counts and biomarker analysis. 

Problems concerning EV particle detection methods originate from the fact that most of the methods cannot distinguish EVs from other biological nanoparticles and may require antibody labelling or antibody capture. One possible solution is to consider indirect indicators, such as total protein-to-particle or protein-to-lipid ratios. Particle number determination methods include flow cytometry [4,5], nanoparticle tracking analysis [6,7], and resistive pulse sensing [8,9,10]. 

Surface-based analytical methods, such as optical surface plasmon resonance [11,12,13] can provide sensitive particle counting. Acoustic surface sensitive detection techniques do not require an optically transparent medium; therefore, the analysis of biological fluids is more feasible. Changes in conditions at the interface between the sensor and the liquid due to molecular/cellular adsorption/desorption or viscosity change all shift the frequency of the oscillating sensor that provides the measurement signal. The oscillating sensor surface can be modified to specifically or non-specifically capture EVs. A quartz crystal microbalance (QCM) with a CD63 EV specific antibody modified sensor has been used to detect EVs from isolates [14]. 

The electromagnetic piezoelectric acoustic sensor (EMPAS) [15,16] operates by remotely induced ultra-high frequency (up to 1.06 GHz) acoustic shear wave generation in the AT-cut quartz sensor crystal. The EMPAS is a highly sensitive method due to the applicable ultra-high frequency overtones. As the EMPAS, unlike the QCM, does not require electrode connections, the homogenous surface of the quartz sensor allows for versatile surface chemistry routes to engineer the surface for a given interaction. EMPAS devices have been used for detection of HIV-2 antibodies in human serum [17], endotoxin of pathogenic *E. coli* in full human blood plasma [18], cocaine [19], breast and prostate cancer metastasis biomarker (PTHrP) [20]. They have also been used in the study of antifouling coating against undiluted goat serum [21], full human blood plasma [22], human serum and bovine milk [23], as well as in the determination of plasmin enzyme of pM concentration levels [24].

The main aim of this study was to investigate the ability of an EMPAS device to detect EVs. For this purpose, a well characterized [25,26] model EV was used: namely, a red blood cell-derived EV. We aimed to follow the non-specific adsorption; therefore, no specific immuno-capture capability has been introduced. Only a clear comparison of interactions with hydrophobic and hydrophilic surfaces has been probed. The results have revealed that a hydrophobic surface has higher binding capacity than a hydrophilic surface in the assayed EV dispersion. A huge increase in the adsorption capacity was observed after one freeze–thaw cycle of the EV sample. This observation shed light not only on the effect of freezing on the integrity of EVs, but also to the possible application of EMPAS devices for the characterization of the purity of EV samples without the need for separation.

## 2. Materials and Methods

### 2.1. Isolation of Red Blood Cell Derived EVs (REVs)

The use of human blood samples was carried out by following the guidelines and regulations of the Helsinki Declaration in 1975, and was approved by the Scientific and Research Ethics Committee of the Hungarian Medical Research Council (ETT TUKEB 6449-2/2015). Human red blood cell-derived EVs (REVs) were isolated according to the protocol described previously [25]. Briefly, 3 × 6 mL whole blood was collected from healthy volunteers with informed consent into vacuum tubes containing EDTA anticoagulant (VACUETTE^®^ TUBE 6 mL K3EDTA, Greiner Bio_One, Mosonmagyaróvár, Hungary). The red blood cells (RBC) were sedimented by centrifugation at 2500 *g* for 15 min (Nüve NF800R), and after the removal of plasma and white blood cells, were resuspended in isotonic saline solution (0.9% NaCl, B. Braun AG) and washed three times (2500 *g* for 10 min at 4 °C). 3.5 mL of washed RBC were diluted to 10 mL with phosphate buffered saline (PBS, pH = 7.4, Sigma-Aldrich and stored at 4 °C for 7 days. REVs were isolated from the RBC suspension via two consecutive centrifugations (1500 *g* for 10 min and 2850 *g* for 30 min). The supernatant was centrifuged at 16,000 *g* for 30 min (Eppendorf 5415R). The final pellet was suspended in total 100 μL PBS, and purified with size exclusion chromatography (SEC) using a 3.5 mL gravity column filled with Sepharose CL-2B gel (GE Healthcare, Sweden). The 100 mL EV sample was pipetted into the column followed by 900 mL PBS. The second 1 mL fraction containing the purified EVs was collected and kept at 4 °C.

### 2.2. Microfluidic Resistive Pulse Sensing (MRPS)

MRPS is based on the Coulter principle realized in a microfluidic cartridge. MRPS measurements were performed with an nCS1 instrument (Spectradyne LLC, Torrance, CA, USA). The samples were diluted 20-fold with a bovine serum albumin (BSA, Sigma-Aldrich, Hungary) solution at 1 mg/mL in PBS buffer (Sigma-Aldrich, Hungary), filtered through an Amicon Ultra 0.5 centrifugal filter with 100 kDa MWCO (Merck-Millipore, Hungary) according to the manufacturer’s instructions. All measurements were performed using factory calibrated TS-400 cartridges with a measurement range from 65 nm to 400 nm.

### 2.3. Fourier-Transform Infrared Spectroscopy (FTIR)

Attenuated total reflection (ATR) infrared spectroscopy was used to obtain signature spectra for protein and lipid composition of isolated REV samples. 3 µL of fresh REV sample was spotted and dried under ambient conditions on the top of the diamond ATR element of a single reflection Golden Gate accessory (Specac Ltd., Orpington, UK) fitted into a Varian 2000 FT-IR spectrometer (Varian Inc., Paolo Alto, CA, USA). Spectra were recorded by coaddition of 64 individual scans with a nominal spectral resolution of 2 cm^−1^. Before the spectrum evaluation, ATR correction and the subtraction of the PBS buffer background were performed. For all spectral manipulations, the GRAMS/32 software package (Galactic Inc, Birmingham, AL, USA) were used.

### 2.4. Size Exclusion Chromatography with On-Line Fluorescence Detection (Flu-SEC)

Flu-SEC was used to study the release of soluble proteins from EVs upon a freeze–thaw cycle. 10 µL of REV sample was injected into a Jasco HPLC system (Jasco, Tokyo, Japan) consisting of a PU-2089 pump with a UV-2075 UV/Vis detector and a FP-2020 fluorescence detector controlled by the Chromnav software v. 1.17.02. Tricorn 5/100 glass columns (GE Healthcare Bio-Sciences AB) were packed with Sepharose CL-2B (GE Healthcare Bio-Sciences AB), and the eluent was PBS with a flow rate of 0.5 mL/min. The fluorescence chromatograms were collected at excitation and emission wavelength corresponding to the intrinsic fluorescence of proteins (280/340 nm), and the area under the curve of the EV peak and the free protein peak was used to quantify the amount of unbound proteins.

### 2.5. Electromagnetic Piezoelectric Acoustic Sensor (EMPAS) Measurements

Hydrophilic (*θ*_a_ = 15°) [24] quartz crystal discs were obtained using the following method. The discs underwent ultrasound cleaning in detergent solution for 30 min in test tubes, then were copiously rinsed with tap water and distilled water, followed by a 30 min treatment in Piranha solution (3:1 *V/V* mixture of 98% H_2_SO_4_ and 30% H_2_O_2_) at 90 °C pre-heated in a water bath. They were then thoroughly rinsed with distilled water and methanol, followed by sonication in another portion of methanol for 2 min, and a final rinse with methanol. Hydrophobic (*θ*_a_ = 106°) [24] discs were obtained by further processing the hydrophilic ones as follows. The cleaned, hydrophilic discs were individually transferred into glass vials, which were subsequently placed in an oven maintained at 150 °C for drying. After 2 h, the vials were immediately transferred into a humidity chamber (70–80% relative humidity, room temperature) for 36 h of surface moisturization. Then, the quartz discs were individually transferred to silanized test tubes and moved into a glove box under nitrogen atmosphere. Portions of 1 mL of octadecyltrichlorosilane (OTS) solution in anhydrous toluene (1 μL OTS/999 μL toluene, corresponding to 2.5 mM) were added individually to the test tubes. The tubes were then sealed with rubber stoppers, removed from the glove box, and allowed to stay on a shaker for 2 h. The quartz discs were then rinsed thoroughly with toluene, followed by chloroform, and dried under a gentle stream of nitrogen before being stored in scintillation vials.

The experiments were run using a home-built electromagnetic piezoelectric acoustic sensor [15]. The setup consisted of a Plexiglas flow-through cell (~78 μL internal volume); a ~5 mm diameter hand-wound coil of a 105 μm diameter polyurethane-coated copper wire (Goodfellow); a frequency generator (Hewlett Packard 8648B); a trimmer (muRata, Seminole, FL, USA) placed in parallel with the coil terminals in order for the coil to be tuned to electrical resonance; a lock-in amplifier (SR510, Stanford Research Systems); a diode detector (made in house) to measure the voltage drop developed across the coil terminals at acoustic resonance, with the output being fed into the lock-in amplifier; a digital oscilloscope (Tektronix TDS 210); and a syringe pump (Harvard Apparatus Pump 11) equipped with a 60 mL plastic syringe (Henke-Sass, Wolf GmbH, Germany) providing a flow rate of 25 µL/min. Teflon tubing (1.58 mm od, 0.8 mm id, Supelco, Bellefonte, PA, USA) was used to connect the syringe, flow-through cell and sample vial. In order to minimize temperature effect on the resonant frequency, AT-cut quartz crystal wafer resonators were used. The crystals (*⌀* = 13 mm diameter, *t* = 83 µm thickness, Laptech Precision Inc., Bowmanville, ON, Canada) were operated at the 49th overtone of their *f*_0_ = 20 MHz fundamental frequency (*f*_49_ ≈ 984 MHz). A code running under LabView 6.0 was used to control the frequency generator and the lock-in amplifier, as well as for data acquisition. REV samples were used in half logarithmic serial dilutions with PBS (pH = 7.4, Sigma-Aldrich), with the dilution factor denoted with *D*, where *D* ≡ −log (*c*/*c*_0_) = [0.5, 1.0, 1.5, 2.0, 2.5, 3.0], *c* being the concentration of the diluted solution and *c*_0_ the original concentration of the sample. Measurements were conducted at 20 °C with REV samples stored at 4 °C after 1 h waiting for thermal equilibration or with REV samples stored at 4 °C, followed by a storage at −30 °C for periods ranging from 1 h to several days, after 1 h waiting for thermal equilibration.

## 3. Results and Discussion

### 3.1. Characterization of EV Samples

**Figure 1:**
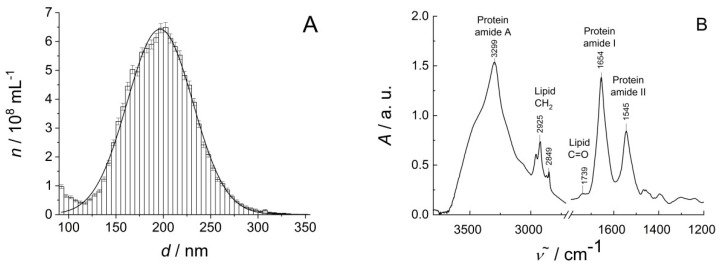
Characterization of red blood cell-derived EVs. (**A**) Particle size distribution of REVs by Microfluidic Resistive Pulse Sensing (MRPS). Number concentration (*n*) of vesicles as a function of the diameter (*d*) (50-fold dilution of the original sample); (**B**) Fourier-transform infrared (FTIR) spectrum of the REV sample and the assignment of the main lipid and protein bands.

Characterization of REVs has been accomplished by MRPS and FTIR methods. The characteristic results are shown in Figure 1. Figure 1A illustrates MPRS measurement results showing that the REVs, prepared as described, are a homogeneous EV sample with well-defined particle size distribution. The results can be well fitted with a Gaussian distribution which results in a mean diameter of (196.9 ± 0.3) nm with standard deviation (32.26 ± 0.3) nm (adj. *R*^2^ = 0.985). The measured concentration is (1.44 ± 0.01) × 10^10^ mL^−1^ over a size range from 65 nm to 400 nm. The FTIR spectrum in Figure 1B indicates the characteristic lipid and protein bands of REVs. Characteristic bands of the peptide backbones of proteins report at 3299 cm^−1^ (amide A, N–H stretching vibrations), 1654 cm^−1^ (amide I, primarily the C=O stretching vibrations of the amide groups) and 1545 cm^−1^ (amide II, combination of N–H bending and C–N stretching of the amide groups). The long acyl chains of lipids exhibit C–H stretching vibrations in the 3020–2800 cm^−1^ wavenumber region with peaks at 2925 and 2849 cm^−1^, corresponding to antisymmetric and symmetric stretching of methylene groups, respectively. The relative weak band at 1739 cm^−1^ belongs to the glycerol carbonyl stretching of the phospholipids. Since the protein-to-lipid ratio can be used to assess EV type and purity [3,27], we calculated the spectroscopic protein-to-lipid ratio [28]. Based on the integrated area of amide I band and C–H stretching bands (3020–2800 cm^−1^) a spectroscopic protein-to-lipid ratio value of 1.52 ± 0.05 was obtained, which is in line with our previous results on purified REV samples [25]. The calculated protein-to-lipid ratio indicates a homogeneous EV sample without impurities. Moreover, as the intensity of the amide I band is proportional with the number of peptide groups, an estimation of the total protein concentration of intact EVs, based on IR spectrum, is also feasible. Using the protocol elaborated by Szentirmai et al. [26], the IR spectroscopy-based total protein concentration of the REV sample is (0.95 ± 0.09) mg/mL.

### 3.2. Effect of Freezing Temperature and the Duration of Frozen Storage of EVs on EMPAS Signal 

**Figure 2:**
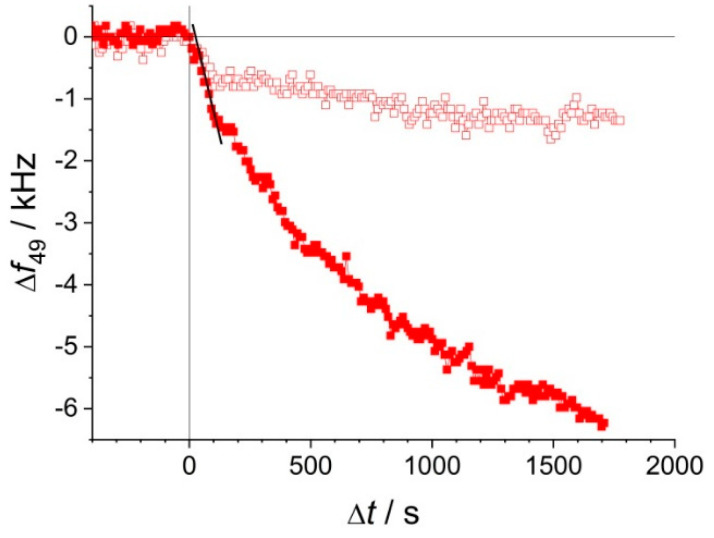
A typical measurement outcome: time course of frequency shift at the 49th overtone of two hydrophobic quartz crystals, under two EV samples of identical dilution, *D* = 0.5 (3.16× dilution of the stock), without freezing (☐), and after a 1 h exposure to −30 °C followed by 1 h thermal equilibration at lab temperature (■). The slope of the thick black fitted line represents the maximum rate of frequency shift, (d*f*/d*t*)_max_, for the second case.

Two typical EMPAS measurements are presented in Figure 2. In both cases, after recording the baseline with PBS only, the EV solution enters the cell at Δ*t* = 0 s, producing a frequency shift of the quartz resonator. The general observation was that the time course of the frequency shift was of a modest rate for EV samples stored at room temperature and 4 °C, even at the relatively high EV concentrations (3.16× dilution of the original sample, *D* = 0.5), whereas the frequency decreased much faster if an identical EV sample had previously been stored at −30 °C, suggesting that freezing might induce structural changes of the EV samples resulting in either an increased number, or a reduced size (i.e., larger diffusion coefficient) of any adsorbates. 

**Figure 3:**
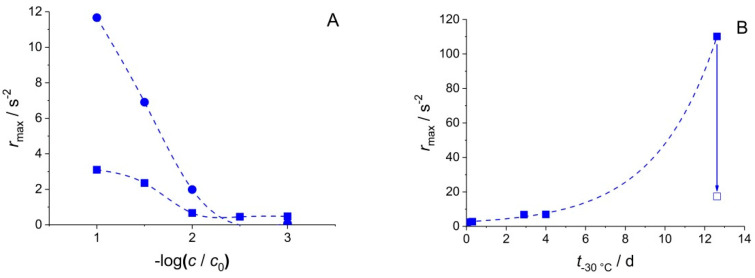
(**A**) *r*_max_ at the 49th overtone on hydrophilic quartz crystals for different dilutions of the REV stock solution, after a 60 min (■) and 4 days (●) exposure, respectively, to −30 °C. (**B**) Exponential dependence of *r*_max_ on the storage time at −30 °C of REV *D* = 1.5 solutions (i.e., 31.6× dilution of the original stock), measured at the 49th overtone on hydrophilic quartz crystals (■). Data point marked with ☐ was measured after at 12.6 d storage at −30 °C, followed by a 4 d storage at +4 °C.

Interestingly, the duration of frozen storage was found to affect the EMPAS signal in a broad REV concentration range spanning three orders of magnitude. Figure 3A,B illustrate the qualitative changes observable as a result of storage temperature dependence. The maximum rate of frequency change, *r*_max_, defined in Equation (1), has been used to express the adsorption rate of the sample components.

(1) $$r_{\max}\overset{\mathrm{def}}{=}-{\left(df/dt\right)}_{\max}$$

The increase of *r*_max_ with EV concentration is not surprising, as *r*_max_ is expected to be a symbatic function of the maximum rate of adsorption, which in turn is expected to increase with increasing mass transport, and therefore, concentration. However, surprising is the markedly different dependence of *r*_max_ on concentration for the samples stored for different time intervals (1 h or 4 d) at −30 °C. The observation suggests that the structural change phenomenon induced by freezing is not instantaneous, but progresses, even at −30 °C, on a timescale of several days. The study of this effect for additional storage time values with identical REV concentrations lead to the result shown in Figure 3B. *r*_max_ shows a clear, nonlinear, probably exponential dependence on the storage time at −30 °C. Figure 3B also illustrates an interesting, and not yet fully understood, phenomenon about the reversibility of long-term storage effect: namely, the storage time dependent adsorption rate increase can be turned back to a lower value by storing the sample in a thawed state for 4 days. This result might indicate a slow, partial re-aggregation of the REV component molecules after thawing, decreasing the concentration, and re-increasing the size (hence decreasing the diffusion coefficient) of the available adsorbates.

The effect of freeze–thaw cycles on EV functionality has been investigated in multiple cases, but the results are quite variable. Kim et al. [29] observed that four freeze–thaw cycles disrupted the integrity of exosomes (from murine bone marrow-derived dendritic cells), and consequently, the immunosuppressive ability of exosomes was lost. In an untreated sample the exosome-associated heat shock protein Hsc70 was found to be associated with the exosomes but not in the freeze–thaw treated exosome fractions. Lőrincz et al. have found that the storage of neutrophilic granulocyte-derived EVs at room temperature or 4 °C for a day did not influence the vesicle count, but induced a loss of function. Sample storage at −20 °C resulted in changed light scattering properties, and the functionality was almost completely lost after 28 days. After storage at −80 °C for 1 month, light scattering properties did not change, but the number and size of vesicles changed slightly, and the functionality was partially lost [30]. The concentration of exosomes from Sprague Dawley rat bone marrow mesenchymal stem cells was reported to lower after freezing to −80 °C and subsequent thawing. The functionality was also affected [31].

**Figure 4:**
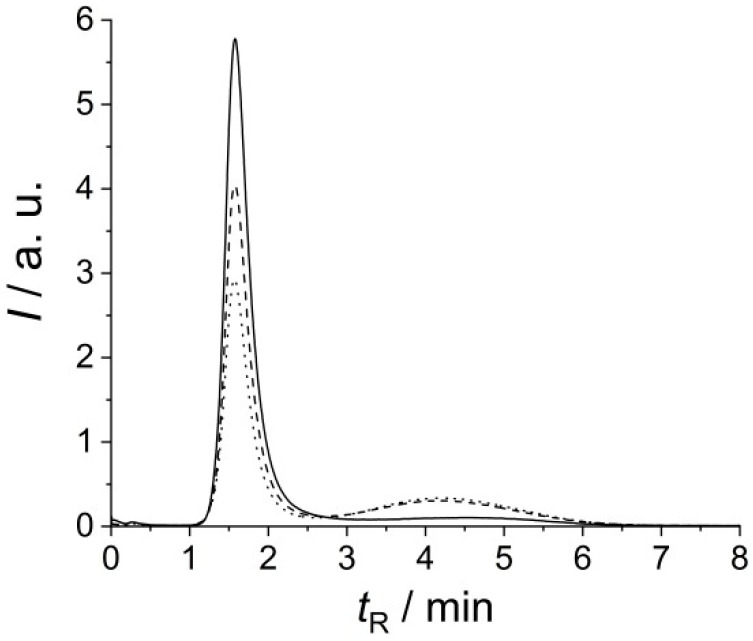
Size exclusion (Flu-SEC) chromatograms (fluorescence intensity *I* versus retention time *t*_R_) of control REVs (solid line), and REVs after one (dashed line) and two (dotted line) freeze–thaw (FT) cycle(s) measured at excitation and emission wavelengths of 280 nm and 330 nm, respectively.

In our case, the changed sample integrity, i.e., protein desorption from the protein corona of the vesicle, might serve as a feasible explanation for the different EMPAS signals in the detection of frozen and non-frozen REVs. To evaluate this possibility, Flu-SEC chromatograms have been measured in consecutive freeze–thaw cycles (Figure 4). Control REVs are eluted at 1.5 min retention time and no sign of free proteins can be observed. Freeze–thaw cycles result in the appearance of a second peak at 4.1 min retention time, which corresponds to free proteins, i.e., to unbound membrane-associated proteins.

**Figure 5:**
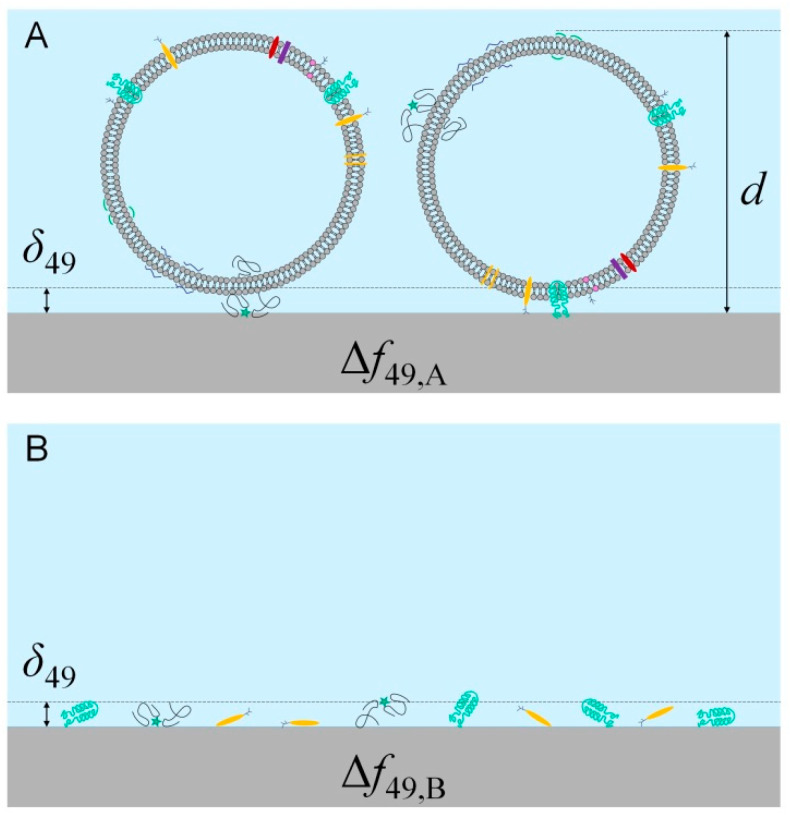
Penetration depth (*δ*) compared to the size of adsorbed entire extracellular vesicles (EVs) (**A**) and EV-proteins (**B**), respectively, approximately to scale. For maximum packing densities, the frequency shift on the 49th overtone is higher if only proteins adsorb: Δ*f*_B_ *>* Δ*f*_A_.

As a sum of observations, we can conclude that intact REVs produce low EMPAS signals, but freezing resulted in disintegration and protein desorption, which appears in a well observable, EV concentration dependent signal. To explain these differences, two effects can be considered: (1) the penetration depth of the acoustic wave, and (2) a large difference in the diffusion coefficients of free protein molecules and EVs. The penetration depth (*δ*) of the acoustic wave is the distance perpendicular to the surface of the resonating quartz crystal which is reached by the 1/*e*-th (~37%) of the energy of the acoustic wave. This distance is a function of the viscosity (*η*) and density (ρ) of the medium, as well as the ground resonance frequency (*f*_0_) of the crystal and the overtone number (*n*) at which it is operated. By approximating the viscosity and density of the PBS solution with that of water (*η* = 10^−3^ Pas, *ρ* = 10^3^ kg/m^3^), and further plugging in *f*_0_ = 20 × 10^6^ Hz and *n* = 49, the resulting estimate is *δ* = 18 nm. Table 1 shows the mean square displacement values of intact vesicles and disintegrated proteins calculated for 180 s time scale, the average duration of stay in the flow cell. By comparing this penetration depth with the mean diameters of EVs (Table 1), it becomes clear that a large frequency shift cannot be obtained if the EVs adsorb as whole, intact globules, not even at high surface coverages; conversely, a large frequency shift is potentially observed if only proteins, or other EV-fragments adsorb, even at lower surface coverages (Figure 5).

(2) $$\delta =\sqrt{\frac{\eta }{\pi \rho nf_{0}}}$$

### 3.3. Quantitative Assay Feasibility Study and Sensor Surface Modification for Optimal Detection with EMPAS

Although the results have shown that EMPAS measurements based on non-specific adsorption are insensitive for EVs, the freezing-detached protein fraction of EV suspension can still be a sensitive, quantitatively correlating target to determine. Very importantly, it may even provide a separation-free way to characterize EV isolations in accordance with MISEV 2018 guidelines.

**Figure 6:**
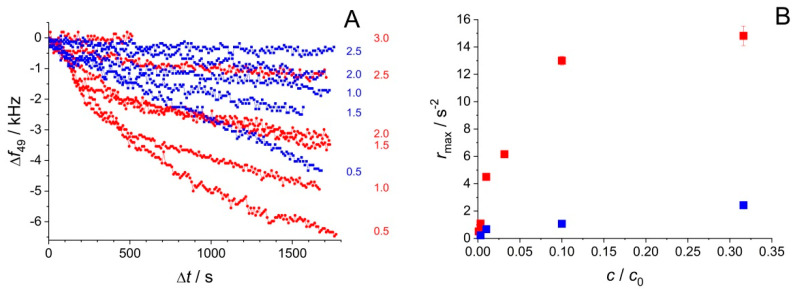
Surface modification effect on the frequency response of electromagnetic piezoelectric acoustic sensor (EMPAS) signal upon adsorption. (**A**) Time course of frequency shift at the 49th overtone on hydrophilic (blue) and hydrophobic (red) quartz crystals, for different dilutions expressed as –log(*c*/*c*_0_) of the EV stock solution, after a short exposure (less than 1 day) to −30 °C. (**B**) *r*_max_ at the 49th overtone on hydrophilic (blue) and hydrophobic (red) quartz crystals, for different dilutions of the REV stock solution, after a short exposure (less than 1 day) to −30 °C.

To optimize the adsorption of the protein fraction, measurements using hydrophilic and hydrophobic EMPAS crystals have been conducted. EV dilution series after one freeze–thaw cycle have been measured (Figure 6).

A comparison of hydrophobic and hydrophilic surfaces shows that for the same EV dilution the EMPAS signal has a greater frequency shift in the case of hydrophobic surfaces, indicating a higher adsorption rate. Although vesicle quantification cannot be directly achieved with the present configuration, the amount of measurable free proteins adsorbed on the crystal correlates to the number of vesicles, and thus the methodology developed may serve as an indirect method for quantification. For this approximation, we use the protein concentration calculated from IR measurements given as *c*_0_ = 950 μg/mL. Based on Flu-SEC measurements, we assume that 30% of the total protein is detached, and as the most diluted sample is −log(*c*/*c*_0_) = 2.5 ≈ 0.3% dilution, the detection limit is in the range of 0.9 μg/mL of protein. The vesicle concentration in the original sample is *c*_REV_ = 5.6 × 10^11^ mL^−1^ according to MPRS measurements, so the detection limit for vesicle concentration in the given conditions is in the range of 10^−9^ mL^−1^. By considering the average size (*r* = 100 nm) and concentration of REVs measured by MRPS and estimating the protein size with the size of an average human protein, we arrive at a realistic approximation of 0.15 protein/nm^2^ of vesicle for the given experimental conditions. It is important to emphasize that the detection limit is valid for the proteins in the EV suspension, hence in the presence of EVs, without separation.

## 4. Conclusions

The adsorption properties of well-characterized red blood cell–derived extracellular vesicles have been investigated with a high sensitivity electromagnetic piezoelectric acoustic sensor. Maximum rates of frequency shifts of the oscillating sensor have been compared to represent differences in adsorption capacities. Freeze–thaw pretreatment of the samples has largely affected the measurable signal, indicating that the disrupted integrity of the vesicles, verified with chromatography, results in well-detectable substances. Hydrophobic surfaces proved to adsorb faster sample content than hydrophilic surfaces did. The phenomenon is well known from the biofouling field: in aqueous environment, low surface energy (hydrophobic) surfaces tend to adsorb faster than high surface energy (hydrophilic) surfaces, especially when the adsorbates are macromolecules, such as (bio)polymers (e.g., proteins). The most plausible explanation is based on the total entropy gain of the system upon releasing the hydrate shell water molecules during the adsorption of the macromolecule. The maximum rates of frequency shifts increased with increasing initial vesicle concentration. As such, this method provides the possibility of indirect vesicle quantification even without separation steps applied. The assay can also be considered as a follow-up technique in sample storage standardization processes.

## Figures and Tables

**Table 1:** Comparison of mean square displacement values of intact vesicles and disintegrated proteins calculated for 180 s time scale, the average duration of stay in the flow cell.

Particle	Radius (nm)	Diffusion Coefficient (m^2^/s)	Mean Square Displacement (µm)
protein	2.5	9.8 × 10^−11^	325.4
vesicle	100	2.5 × 10^−12^	51.4
